# The Need for Evolutionarily Rational Disease Interventions: Vaccination Can Select for Higher Virulence

**DOI:** 10.1371/journal.pbio.1002236

**Published:** 2015-08-25

**Authors:** Mike Boots

**Affiliations:** 1 Integrative Biology, University of California Berkeley, Berkeley, California, United States of America; 2 Biosciences, University of Exeter, Penryn Campus, Penryn, United Kingdom

## Abstract

There is little doubt evolution has played a major role in preventing the control of infectious disease through antibiotic and insecticide resistance, but recent theory suggests disease interventions such as vaccination may lead to evolution of more harmful parasites. A new study published in *PLOS Biology* by Andrew Read and colleagues shows empirically that vaccination against Marek’s disease has favored higher virulence; without intervention, the birds die too quickly for any transmission to occur, but vaccinated hosts can both stay alive longer and shed the virus. This is an elegant empirical demonstration of how evolutionary theory can predict potentially dangerous responses of infectious disease to human interventions.

## Abstract 2

New evidence from studies of Marek’s disease supports evolutionary theory, which suggests that vaccination can select for increased parasite virulence.

There is little doubt that evolution continues to play a major role in preventing drug and vector-control programs from eliminating many infectious diseases. How much of the global infectious disease burden is attributable to recent evolution, and how much to social and other forces, remains unclear, but we are unquestionably severely impacted by the evolutionary potential of pathogens [1]. There is a large body of evolutionary theory that seeks to understand the processes that make some infectious diseases acute and lethal while others are chronic and mild [2–5]. More recently, this general theory has been applied to make predictions of the evolutionary outcomes of particular disease interventions within the broader aim of “virulence management” [6,7,9]. Of particular importance is that the theory predicts that there is the potential for certain disease interventions, including certain types of vaccination, to select for the evolution of greater virulence (cause higher mortality) and therefore present a greater threat to their hosts [7]. However, the theory generally makes deliberately simple assumptions, ignoring, for example, the molecular mechanisms that underpin host–parasite interactions. While this is one of the strengths of the approach—since it aims to make general predictions—it has been unclear how relevant this theory is to real infectious diseases. Read et al. have now provided a direct empirical test of one of the key theoretical predictions that “imperfect” vaccination can select for higher virulence [8]. The study is important because although there is increasing interest in evolutionary biology by the medical community [10,11], few empirical tests of evolutionary theory have been conducted that are of immediate relevance to important disease problems. Importantly, this empirical paper confirms the unintuitive and worrying predictions of a very simple theoretical model of the implications of a common disease intervention.

In some sense, theory on the evolution of virulence addresses the fundamental question of why infectious diseases kill their hosts. In a classic infectious disease, whether spread by contact, environmental infectious stages, or through vectors, the longer the host is infectious, the greater the chance that transmission will occur. If by killing the host the infectious period is shortened, then all things being equal, parasite genotypes that kill the host more slowly have a longer infectious period and will therefore be favored. Hence, virulence (defined as disease-induced mortality) will be selected against by evolution, and parasites should evolve to become benign; they should evolve away from parasitism towards commensalism (infection without host damage). This is the “conventional wisdom” [3] that leads ultimately to the question: why are some parasites lethal? High virulence in a relatively rare host into which a disease occasionally spills over, such as Ebola in humans, may persist because selection is predominately occurring in the reservoir rather than the rare host. Furthermore, recently emerged, initially virulent disease may be in the process of evolving to lower virulence as they become more endemic in a new host. Also, in principle, disease-induced mortality could be a by-product of infection that is completely unrelated to both the genotype and life history characteristics of the parasite and is therefore not selected against. However, evolutionary theory assumes that virulence has been selected for because fundamentally things are not equal. Specifically, most theory assumes that disease-induced mortality (virulence) results from a “trade-off” (a gain in one trait comes at the expense of another) with another parasite characteristic, so that virulence is a correlated and unavoidable consequence of another factor that increases the chance of transmission. In this “trade-off hypothesis,” people have generally focused on virulence (mortality rate) being a by-product/cost of the transmission rate [2,5,12], as it is an appealing idea that high growth rates within the host may produce more transmission stages and therefore a higher rate of transmission, but also cause more damage and therefore higher virulence. Clearly within-host dynamics are much more complicated than this caricature, and we rarely understand all the mechanisms that underpin any trade-offs, but there is now good evidence for this overall relationship between transmission rate and mortality rate (virulence) in a number of systems [5,13–16]. Given this trade-off relationship, the theory predicts that there is an optimal transmission rate and level of virulence that maximizes the average number of infections that would occur in a completely susceptible population (the so called basic reproductive number, R_0_). With any disease intervention, however, there is a clear and present danger that the balance between transmission rate and virulence will be altered, leading to changes in “optimal” virulence. It is here that evolutionary theory can be useful in predicting the impact on infectious disease virulence of different interventions.

The theoretical paper that predicted the empirical results tested in the Read paper was inspired by the potential use of “imperfect” or “leaky” vaccines for malaria [7]. The key assumption of the model is that vaccination is “leaky” such that transmission can occur from infected, vaccinated individuals. If, on the other hand, the vaccination is “sterilizing,” preventing the infection (or at least the infectivity) of vaccinated individuals, then vaccinated individuals represent an evolutionary dead-end for parasites, as there is no opportunity for selection to occur. The model is simple in that it explicitly excludes escape mutants (which in some sense also make vaccines imperfect) and is typical of the approach used in evolutionary theory. The power of the approach is that by focusing on one process, the theory can make clear predictions. The key message of the detailed modeling is that leaky vaccination, which reduces the impact of the disease and thereby lowers pathogenicity, selects for a higher growth rate in the parasite, leading to a greater transmission rate and higher virulence [7]. Effectively, the cost of higher exploitation is reduced, which changes the shape of the virulence-transmission rate trade-off and allows for higher optimal growth and transmission rates. An important consequence is that a highly virulent parasite strain that kills its host so quickly that it cannot persist in an unvaccinated population can potentially circulate in a vaccinated population.

Read et al. present clear evidence that imperfect vaccines do indeed enable the persistence of much more virulent strains of Marek’s disease to circulate than would be possible in the absence of vaccination. Marek’s is a disease of poultry that is spread by inhalation, persists in the environment, and initially causes paralysis in older birds. Previous work from the group had shown clearly that there is a transmission-virulence trade-off in the disease [15,16]. Leaky vaccination [17] against Marek’s has been common since the 1970s, and over this period the disease has become much more virulent [12]. While during this time there have been a number of changes including the intensification of production [18] and shorter bird life spans [15] that could, in theory, have increased virulence, the Read paper directly examines whether leaky vaccines could be the cause. In the new paper, the vaccine is confirmed to be “leaky” [17]: vaccinated birds can become infected and, critically, they can shed the virus. In the core experiment, Read et al. vaccinated birds from naïve parents (so that there were no maternal antibodies) with five virus strains that vary in virulence from 60% mortality over two months to 100% mortality by 10 days. In terms of classic virulence measures, this approximates as a 10-fold variation in the disease-induced mortality rate. Vaccination does reduce shedding of the virus; however, this positive effect of the intervention is overwhelmed in the more virulent viruses by the fact that unvaccinated birds die much more quickly. Without vaccination, the virulent strains generally kill the hosts before any transmission can take place: a strong example of the transmission virulence trade-off in action. The researchers went further and directly examined transmission using sentinel birds. These sentinel birds were put in enclosures with either vaccinated or unvaccinated birds, both of which had been challenged with the more virulent viruses. Early death in the unvaccinated birds meant that no sentinels were infected, and this contrasted starkly with the vaccinated birds enclosure, where the sentinel birds were infected. As a whole, this paper provides a direct test of the idea that vaccination allows the transmission of virus strains that are too virulent to transmit in non-vaccinated hosts.

A key criticism of the Gandon et al. theoretical paper is that there is no clear evidence of higher virulence due to human vaccination programs. However, the established successful human vaccination programs have mostly been “sterilizing” [19], although as we implement human vaccination programs with “leaky” vaccines, we will be carrying out real-world “experiments” that test the theory. The Read paper has shown, however, that this piece of evolutionary theory is pertinent to real-world infectious disease control. More generally, this study highlights the potential usefulness of evolutionary theory for disease control and suggests that it may therefore have an important role to play in the design of medical interventions. If so, it is important that we take a broad view of the evolution of virulence theory and the “trade-off hypothesis” beyond the simple relationship between transmission and mortality rates. Evolutionary theory is directly applicable whenever virulence is an optimum determined by the relative costs and benefits of a number of correlated parasite traits. This broader view is important since, for infectious agents that are obligate killers (i.e., they can only transmit at the death of their host), virulence is positively related to transmission. However, in these diseases there are other trade-offs, such as one between productivity and time to death, that lead to an evolutionarily optimal virulence that is determined by selection [5,20,21]. Indeed, the paper that is often cited as the origin of the trade-off hypothesis [4] described a trade-off between virulence (disease-induced mortality) and recovery (rather than transmission) such that faster growing, more damaging parasite strains are harder to clear, and the hosts take longer to recover [4,22]. Some of the criticism for the trade-off hypothesis focuses on whether there is a transmission–virulence relationship in a particular disease interaction; but if we take this broad view, there is considerable evidence that virulence is shaped by selection [5,12,23]. Furthermore, although “accidental” high virulence in a rare host may not be selected against, selection is likely to be happening in the more common hosts, and even if virulence is caused primarily by host immunopathology, this has the potential to select the parasites to modulate growth or immunomodulation [24–27].

In terms of vaccination programs, it would clearly be useful if there were more experimental tests of the theory. However, there are likely to be few systems in which there has been the widespread historical implementation of leaky vaccination that are also amiable to experimentation. There are, however, a number of key issues that still need to be addressed theoretically. In particular, any vaccination program is likely to have incomplete vaccine coverage, and understanding the impact of different levels of heterogeneity in vaccine coverage within the population to the evolution of virulence is a difficult but important problem. Furthermore, there is likely to be genetic variation both in resistance of hosts and the efficacy of vaccination within most populations, and this heterogeneity may have important implications to the outcome of vaccination [28]. It is also the case that there can often be specificity between different parasite strains and host genotypes, and this may be of considerable importance in many systems [12], particularly outside of relatively genetically homogenous agricultural populations. That said, given that we now have this test in Marek’s disease, we are now able, at least, to say that the theory is relevant to real-world disease systems. It seems prudent, therefore, to take this risk seriously and consider the potential for selection in the use of new vaccines. The theory tells us that the key questions we need to ask of a vaccination program are: is the vaccine leaky? Does the vaccine act to reduce the impact of the disease within an individual? And is the virulence of the parasite selected for—whether it is due to the transmission virulence trade-off or some other, broader trade-off relationship? These questions should ideally be addressed before the implementation of any vaccination program, and careful monitoring would be usefully implemented in the light of this potential selection for higher virulence.

More broadly, other disease interventions beyond vaccination also have the potential to select upon disease and cause similar problems [6]. It is very hard for us to predict the evolutionary outcome after the emergence of a new disease into a population, but we can and should do much better in predicting the impact of our own disease interventions. If we take it seriously, evolutionary theory gives us the opportunity to move towards a more evolutionarily rational program of disease intervention. While the Read paper shows how the simple theory of the Gandon paper can predict real disease dynamics, there is considerable potential for the development of more disease-specific theory that includes more of the key detailed mechanistic knowledge of a particular host–parasite interaction. There is an opportunity for real advances in the predictive power of the models through tight collaborations between evolutionary modelers and molecular parasitologists and/or virologists. Calls for more serious acceptance of evolutionary biology by the medical community are increasing [10,11], and the Read paper shows that a combination of predictive theory and empirical tests of this theory in real-world disease systems have real potential to improve disease interventions in the light of evolutionary responses.
